# Cost-effectiveness of a two-layer compression bandage versus standard bandage following total knee arthroplasty

**DOI:** 10.1302/2633-1462.57.BJO-2023-0153.R1

**Published:** 2024-07-05

**Authors:** Sarah J. Ronaldson, Elizabeth Cook, Alex Mitchell, Caroline M. Fairhurst, Mike Reed, Belén C. Martin, David J. Torgerson

**Affiliations:** 1 Department of Health Sciences, York Trials Unit, University of York, York, UK; 2 Northumbria Healthcare NHS Foundation Trust, Ashington, UK

**Keywords:** Knee, Orthopaedic surgery, Randomized controlled trial, Cost-effectiveness, Economic evaluation, Compression bandage, Knee replacement, Arthroplasty, total knee arthroplasty (TKA), knee arthroplasty, randomized controlled trial, EQ-5D-5L, Sensitivity analysis, Oxford Knee Score, knee, general practitioner, standard deviation, physiotherapist

## Abstract

**Aims:**

To assess the cost-effectiveness of a two-layer compression bandage versus a standard wool and crepe bandage following total knee arthroplasty, using patient-level data from the Knee Replacement Bandage Study (KReBS).

**Methods:**

A cost-utility analysis was undertaken alongside KReBS, a pragmatic, two-arm, open label, parallel-group, randomized controlled trial, in terms of the cost per quality-adjusted life year (QALY). Overall, 2,330 participants scheduled for total knee arthroplasty (TKA) were randomized to either a two-layer compression bandage or a standard wool and crepe bandage. Costs were estimated over a 12-month period from the UK NHS perspective, and health outcomes were reported as QALYs based on participants’ EuroQol five-dimesion five-level questionnaire responses. Multiple imputation was used to deal with missing data and sensitivity analyses included a complete case analysis and testing of costing assumptions, with a secondary analysis exploring the inclusion of productivity losses.

**Results:**

The base case analysis found participants in the compression bandage group accrued marginally fewer QALYs, on average, compared with those in the standard bandage group (reduction of 0.0050 QALYs (95% confidence interval (CI) -0.0051 to -0.0049)), and accumulated additional mean costs (incremental cost of £52.68 per participant (95% CI 50.56 to 54.80)). Findings remained robust to assumptions tested in sensitivity analyses, although considerable uncertainty surrounded the outcome estimates.

**Conclusion:**

Use of a two-layer compression bandage is marginally less effective in terms of health-related quality of life, and more expensive when compared with a standard bandage following TKA, so therefore is unlikely to provide a cost-effective option.

Cite this article: *Bone Jt Open* 2024;5(7):550–559.

## Introduction

Total knee arthroplasty (TKA) is a common surgical procedure for the treatment of knee pain and disability caused by osteoarthritis, rheumatoid arthritis, and post-traumatic arthritis. Swelling of the knee often occurs postoperatively, causing pain and reduced knee function, which can result in increased lengths of hospital stay, delayed rehabilitation, and reduced patient satisfaction^1^; hence representing a considerable burden to patients and costs to the health service. Methods to address these issues include use of a cold compress, cryotherapy, elastic bandaging, and compression bandaging. Although established evidence is available for use of a compression bandage system for other indications, namely the treatment of venous leg ulcers and lymphoedema,^2,3^ it had not been robustly investigated for use following TKA. Data from a feasibility study of an inelastic, short-stretch compression bandage following TKA indicated it to be a safe technique and acceptable to patients, with potential for improving patient reported health outcomes.^4^ The Knee Replacement Bandage Study (KReBS) was therefore undertaken to evaluate the effectiveness and cost-effectiveness of a two-layer compression bandage versus standard wool and crepe bandage following TKA.

KReBS found there to be no improvement in the primary outcome, the Oxford Knee Score (OKS)^5,6^ at 12 months; the mean OKS at 12 months post-randomization was 36.4 (standard deviation (SD) 10.3) and 36.0 (10.3) for the compression and standard bandage groups, respectively, with an adjusted mean difference of 0.29 (95% confidence interval (CI) -0.60 to 1.20; p = 0.519). The full clinical effectiveness results will be reported separately. Regardless of the lack of a statistically significant clinical effect, it remains important to assess the impact of compression bandaging in terms of patients’ health-related quality of life (HRQoL) and healthcare resource use. The economic evaluation conducted using patient-level data collected from KReBS to determine the cost-effectiveness of compression bandages versus standard bandages following TKA is presented in this paper.

## Methods

### Population and trial details

KreBS was a pragmatic two-arm, open label, parallel-group, randomized controlled trial conducted in 26 UK NHS hospitals, with the primary outcome of the OKS at 12-month follow-up, to represent change in knee function. Patients scheduled for primary TKA aged 18 years and over were included in the study and randomized on a 1:1 basis to receive either a two-layer compression bandage (Coban 2; 3M, UK) or a standard wool and crepe bandage (i.e. the control group). Full details of the trial design and clinical effectiveness results are available elsewhere.^7^

### Ethical approval

The study (ISRCTN 87127065) had ethical approval provided by Newcastle & North Tyneside 2 Research Ethics Committee (16/NE/0400).

### Data collection

Data were collected by means of participant self-completed questionnaires at baseline and 12-month follow-up, and also via NHS Digital’s patient-reported outcome measures (PROMs) project and local patient administration system (PAS) data from study sites (provided by 66% of sites). Specifically, for the economic analysis, HRQoL, resource use, and cost data were obtained using participant questionnaires; PAS data obtained from study sites were used for initial length of hospital stay, complications, comorbidities, and demographics; adverse event forms were used to capture complications; and additional EuroQol five-dimension (EQ-5D) data were obtained via PROMs.

### Analysis overview

The economic evaluation took the form of a cost-utility analysis, which evaluated the compression bandage versus standard bandage in terms of the incremental cost per quality-adjusted life year (QALY). The base case analysis adopted the perspective of the UK NHS and Personal Social Services (PSS), including healthcare services used by participants over the 12-month follow-up period in relation to their knee arthroplasty and the costs of the bandaging systems. A secondary analysis explored the broader societal perspective by also considering indirect costs of lost productivity. Analyses used multiple imputation, with a complete case analysis also undertaken. Intention-to-treat principles were followed and analyses were conducted over a 12-month period, therefore discounting of costs and health outcomes was not necessary. Costs are presented in pounds sterling (£) for 2018 to 2019, with the NHS cost inflation index used where required to inflate costs to this year of pricing.^8^ Sensitivity analyses explored the impact of altering key assumptions in terms of the overall findings. All analyses were conducted using Stata version 16 (StataCorp, USA).

### Resource use and costs

Healthcare resource use data were collected for each participant over a 12-month period using self-completed participant questionnaires at 12 months post-randomization. Consultations occurring within primary care and the community (general practitioner (GP), nurse, physiotherapist, occupational therapist visits) and the hospital setting (outpatient attendances, day case visits, inpatient nights, and emergency department (ED) attendances) were recorded. Participants were asked to record resource use relating to their knee arthroplasty and also resource use relating to any ‘other reason’. Only knee arthroplasty-related resource use was included in the base case analysis. As no statistically significant difference was found between the groups in terms of resource use relating to any other reason, no further exploration via sensitivity analysis was undertaken, as set out in the health economics analysis plan. The base case assumed that any resource use relating to complications was captured through participants’ responses to resource use questions in the participant questionnaires. The total cost per participant was calculated by multiplying the resources used by the corresponding unit costs. Unit costs of healthcare resource use items (Table I) were sourced from established costing sources comprising NHS Reference Costs^9^ and PSSRU Unit Costs of Health and Social Care.^8^ In addition to healthcare use, the base case analysis included costs associated with the bandage used (see ‘bandage costs’ for details). A secondary analysis undertaken from a societal perspective considered costs regarding lost productivity, specifically days missed from work. Productivity loss over the 12-month period was estimated by multiplying the total number of missed workdays self-reported by participants by a daily wage of £114 (based on weekly earnings of £569).^10^

**Table I.** Baseline characteristics of study population.

Variable	Compression bandage (n = 1,213)	Usual care (n = 1,117)	Overall (n = 2,330)
**Sex, n (%)**			
Male	534 (44.0)	509 (45.6)	1,043 (44.8)
Female	677 (55.8)	605 (54.2)	1,282 (55.0)
Missing	2 (0.2)	3 (0.3)	5 (0.2)
**Age, yrs**			
n (%)	1,211 (99.8)	1,115 (99.8)	2,326 (99.8)
Mean (SD); range	68.8 (8.8); 42.4 to 93.3	69.2 (9.1); 42.4 to 92.8	69.0 (8.9); 42.4 to 93.3
Median; Q2 to Q3 (IQR)	69.2 (62.7 to 75.0)	69.5 (62.8 to 75.7)	69.3 (62.7 to 75.2)
**BMI, kg/m** ^**2**^			
n (%)	1,182 (97.4)	1,093 (97.9)	2,275 (97.6)
Mean (SD); range	31.3 (5.6); 14.3 to 58.3	31.5 (5.4); 19.0 to 35.0	31.4 (5.5); 14.3 to 58.3
Median; Q2 to Q3 (IQR)	30.8 (27.3 to 34.8)	31.0 (27.9 to35.0)	30.9 (27.6 to 34.9)
**Knee receiving treatment, n (%)**			
Left	451 (37.2)	412 (36.9)	863 (37.0)
Right	523 (43.1)	450 (40.3)	973 (41.8)
Missing	239 (19.7	255 (22.8)	494 (21.2)

IQR, interquartile range; SD, standard deviation.

### Bandage costs

Costs associated with the bandage system comprised staff time for bandage application and removal and the cost of the bandage itself. In addition, training was provided for the surgical staff involved in applying the compression bandage; therefore, a training cost was applied for participants who received this type of bandage. The different bandage cost components for both groups are shown in Table II. For training on compression bandage application, information provided by study personnel was used to assume that: each trainee received ten minutes’ training (watching a training video, reading an instruction leaflet, and discussion with other team members); the principal investigator at each site received ten minutes’ support from the trainer; and there were six trainees per site for each of the 26 sites. Costs of the relevant healthcare professionals’ time (Table II), for both trainees and trainers, were attached to the time spent on training, to generate a total training cost. This was divided by the number of participants who received the compression bandage to generate a training cost per participant, and applied to each participant who received a compression bandage.

**Table II.** Unit costs associated with bandages.

Item	Unit cost (£)	Additional notes	Source
Compression bandage	8.40	Coban (3M, UK) two multi-layer compression bandage kit 10 cm x 3.5 m.	BNF^11^
Standard bandage	1.56	Velband bandage 10 cm x 4.5 m (BSN Medical, Germany) £0.75 + Hosicrepe 239 bandage 10 cm x 4.5 m (Paul Hartmann, Germany) £0.81.	BNF^11^
Training for compression bandage application	2.46	Based on ten minutes’ training received per trainee, plus ten minutes’ of PI support, both provided by trainer; cost per hour of £64.50* for trainee, £109 for PI (consultant surgeon), and £78 for trainer.*	PSSRU,^8^ KReBS study team
Application of compression bandage	5.02	Based on 4.7 minutes† at a cost of £64.50 per working hour.‡	PSSRU,^8^ KReBS study team
Application of standard bandage	2.51	Based on 2.3 minutes† at a cost of £64.50 per working hour.‡	PSSRU,^8^ KReBS study team
Removal of compression bandage	2.13	Based on 3.0 minutes† at a cost of £42.50 per working hour.§	PSSRU,^8^ KReBS study team
Removal of standard bandage	1.06	Based on 1.8 minutes† at a cost of £42.50 per working hour.§	PSSRU,^8^ KReBS study team

* Based on average of the hourly cost of a consultant surgeon (£109) and registrar (£47).

† Average time taken from study nurses/investigators.

‡ Based on average working hour cost of the surgical team members involved in bandage application: surgical consultant (£109), registrar (£47), and surgical practitioners of band 6 (£47) and 7 (£55).

§ Based on average working hour cost of staff involved in bandage removal: band 5 hospital-based nurse (£38) and band 6 hospital-based nurse specialist (£47).

BNF, British National Formulary; KReBS, Knee Replacement Bandage Study; PI, principal investigator; PSSRU, Personal Social Services Research Unit.

Estimates of time spent applying and removing bandages, provided by taking an average of responses given by study healthcare staff, were multiplied by the unit cost for the relevant healthcare professional’s time, as detailed in Table II. Bandages were removed between 24 and 48 hours after application. Where bandages were removed less than 24 hours after application and there was relevant information recorded, an additional cost of application and removal was included if bandages were re-applied; study notes were used to attach a cost for the particular bandage that was used for the second application.

### Outcome data

Participants’ HRQoL represented by utilities were derived from their EuroQol five dimension five-level (EQ-5D-5L)^12^ questionnaire responses, collected at baseline and 12 months using self-completed questionnaires. Responses provided for the five domains of the EQ-5D-5L generate a specific health state, where full health is represented by a utility score of one, death is represented by a score of zero, and negative scores indicate states worse than death. Following UK National Institute for Health and Care Excellence (NICE) guidance regarding use of EQ-5D-5L data for analyses,^13^ utilities have been estimated using the mapping function developed by van Hout et al.^14^ Regression methods explored the difference in EQ-5D-5L index scores between the two groups, including covariates consistent with those included in the primary statistical analysis model. Utilities were converted into QALYs using the area under the curve approach,^15,16^ and the difference in QALYs between groups over the 12 months was adjusted for baseline utility,^17^ hence allowing for any baseline differences between the groups. In addition to the EQ-5D-5L data, further EQ-5D data were collected to enable a comparison of responses obtained from the EQ-5D-5L and EQ-5D-three level questionnaire (3L).

### Missing data

Multiple imputation (MI) was used to deal with missing data; MI generates unbiased, plausible estimates for the missing data, dependant on the assumption of the data being missing at random. Logistic regression was used to investigate the plausibility of the missing at random assumption, by exploring the association between missingness (of costs and QALYs) and baseline covariates and observed outcomes. With the exception of the complete case analysis, all analyses used multiple imputation with chained equations (MICE) with predictive mean matching on QALY and cost estimates, thereby ensuring plausible imputation values. The imputation model included age, sex, baseline OKS, study site, utilities (at baseline and 12 months), and total costs at the resource use level. A total of 40 imputations were undertaken for the base case, following guidance that the number of imputed datasets should be at least greater than,^18^ or similar to,^19^ the proportion of missing data, which was 38% in the base case. This guidance was followed similarly for all sensitivity analyses. Imputed datasets were combined following Rubin’s rules to generate mean cost and QALY estimates.^20^ Usual imputation methods were followed for participants who died during the study, for any missing data arising before their death. For data that would have been gathered after their death, zero QALYs and resource use were assumed. A complete case analysis was undertaken for comparison, which relies on the data being not missing at random, with available case analysis used for initial investigation of the data.

### Data analysis

Mean incremental costs and QALYs for the compression versus standard bandage groups were estimated using seemingly unrelated regression for 10,000 replications, while adjusting for baseline covariates (age, sex, study site, OKS score, and baseline utility), with 95% CIs estimated using bias corrected and accelerated bootstrap methods. Incremental cost-effectiveness ratios (ICERs) were used to express cost-effectiveness, where appropriate, by dividing the incremental costs by the incremental QALYs. A treatment option is found to be dominated if it is more costly and generates fewer QALYs than the alternative. The current threshold of £20,000 to £30,000 per QALY recommended by NICE has been used for the analyses.^21^ Results were also presented in terms of incremental net monetary benefit (NMB),^22^ using the cost-effectiveness threshold of £20,000 to translate the health benefits into monetary terms. An intervention is deemed cost-effective if the mean incremental NMB is positive. Cost-effectiveness acceptability curves (CEACs) represent uncertainty around the findings.

### Analysis of uncertainty

Sensitivity analyses were undertaken to explore the uncertainty around the cost-effectiveness results, while controlling for covariates. Sensitivity analysis (SA)1 comprised a complete case analysis where only participants with complete economic data profiles were included, thereby exploring the impact of excluding participants with missing data. Costs for complications based on PAS data and adverse event forms were included in SA2; specifically study sites provided PAS information about whether participants had encountered myocardial infarction, urinary tract infection, stroke, transient ischaemic attack, thrombocytopenia, pneumonia, or readmission to hospital (all within 30 days of TKA), or a pulmonary embolism or deep vein thrombosis (within 60 days of TKA). SA3 used alternative costs for inpatient nights, outpatient attendances and day case attendances, using the average for all Health Resource Group (HRG) codes, rather than only those that related to knee arthroplasty, as used in the base case (Table III). The cost of initial inpatient stay for TKA surgery using PAS length of stay data was included in SA4. For both SA2 and SA4, the relevant costs for each of the HRG codes reported by PAS data were sourced from NHS Reference Costs and applied accordingly.^9,23^

**Table III.** Unit costs of healthcare services.

Item	Unit cost (£)	Additional notes	Source
Doctor appointment at GP practice	39.23	Per GP visit at surgery of 9.22 minutes duration (including direct care staff costs and qualifications).	PSSRU^8^
Doctor home visit	100.62	Per home GP consultation, comprising 11.4 minute appointment and 12 minutes travel time,^24^ based on £4.30 per minute of patient contact.^8^	PSSRU^8,24^
Doctor phone appointment	30.53	Per phone contact of 7.1 minutes duration^24^ at a cost of £4.30 per minute of patient contact.	PSSRU^8,24^
Nurse appointment at GP practice	10.85	Per nurse visit at surgery lasting 15.5 minutes^24^ at a cost of £42 per hour.^8^	PSSRU^8,24^
Community nurse home visit	25.90	Per home visit, comprising 25 minute appointment^25^ plus 12 minutes travel time,^24^ based on £42 per hour.^8^	PSSRU^8,24,25^
Occupational therapist visit	48.00	Per visit for a community occupational therapist (local authority), including training.^8^ Duration of one hour per visit.^26^	PSSRU^8,26^
Physiotherapist visit	49.50	Based on cost per hour of £49.50, which is the average cost per working hour for physiotherapists of band 5, 6, 7 and 8a.^8^ Duration of one hour per visit.^26^	PSSRU^8,26^
Inpatient night in hospital	426.69	Total HRGs sheet: sum of total expenditure on excess bed days for codes relevant to knee replacement* (elective and non-elective) divided by total activity.	NHS Reference Costs^23^
Day case attendance	1635.01	Total HRGs sheet: sum of total cost divided by total activity for all day cases related to knee replacement.*	NHS Reference Costs^9^
Hospital outpatient appointment	146.51	Total outpatient attendances sheet: rheumatology (code 410).	NHS Reference Costs^9^
ED attendance	166.05	Total HRGs sheet: total activity for all ED attendances divided by total activity, to generate average cost for ED attendances.	NHS Reference Costs^9^

* HRG codes relevant for knee arthroplasty comprise: HN22D, HN22E, HN23B, HN23C, HN24B, HN24C, HN25A, HN26A.

ED, emergency department; GP, general practitioner; HRG, Health Resource Group; PSSRU, Personal Social Services Research Unit.

## Results

**Fig. 1:**
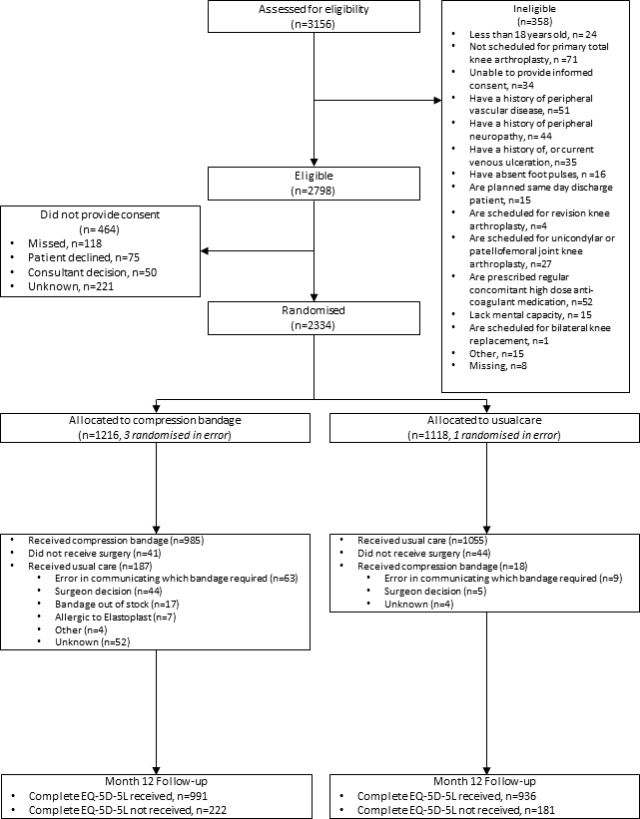
Study flow diagram. EQ-5D-5L, EuroQol five-dimension five-level questionnaire.

A total of 2,330 participants were randomized (four were randomized in error); 1,213 were randomized to receive a compression bandage and 1,117 to receive a standard bandage (Figure 1). Participants were a mean age of 69 years (42 to 93), with 1,282 (55%) participants female (baseline characteristics are summarized in Table I). Overall, 25 participants died during the trial, using predominantly PAS data: 1.32% (16/1213) in the compression bandage group and 0.81% (9/1,117) in the standard bandage group. There were 205 crossovers (187 allocated to the compression bandage group received a standard bandage, and 18 allocated to the standard bandage group received a compression bandage). Crossovers were taken into account in the estimation of bandage costs; for the application, removal, training, and cost of the bandage itself, costs were applied according to the bandage received, irrespective of treatment group allocation. A total of 85 participants did not receive any surgery and therefore did not receive a bandage.

Participants with complete responses for all utility and cost items were categorized as complete cases and included in the complete case analysis (SA1). Complete economic profiles were available for 1,437 (61.7%) participants (757 (62.4%) in the compression bandage group and 680 (60.9%) in the standard bandage group). Logistic regression of missingness found baseline utility and age to be significant predictors of missing QALY data at 12 months. Age and baseline OKS score were significantly associated with missing cost data at 12 months. Data were assumed to be missing at random, with MICE used to deal with missing data for the analysis.

### Resource use and costs

The healthcare services used by participants in relation to their knee arthroplasty are presented in Table IV for all available cases. The proportion of responses missing for each of the resource use items was found to be between 19% and 29%. Use of healthcare services was similar for both groups, with participants most commonly accessing healthcare services for physiotherapist visits, hospital outpatient appointments, and inpatient stays in hospital. The largest mean cost differences between the study groups (compression minus standard bandage), based on the multiply imputed dataset, were seen for hospital day case attendances (£45.24), inpatient nights spent in hospital (£11.19), and outpatient appointments (-£13.17) (Table V).

**Table IV.** Mean healthcare resource use, based on all available cases.

Type of resource use	Compression bandage (n = 1,213)		Standard bandage (n = 1,117)	
	Mean (SD)	Missing, n (%)	Mean (SD)	Missing, n (%)
Doctor appointment at GP practice	0.84 (1.87)	282 (23.3)	0.84 (1.89)	278 (24.9)
Doctor home visit	0.07 (0.53)	268 (22.1)	0.06 (0.36)	267 (23.9)
Doctor phone appointment	0.28 (0.99)	295 (24.3)	0.26 (1.12)	286 (25.6)
Nurse appointment at GP practice	0.34 (0.87)	318 (26.2)	0.51 (2.26)	319 (28.6)
Community nurse home visit	0.45 (1.31)	266 (21.9)	0.52 (1.60)	272 (24.4)
Occupational therapist visit	0.26 (0.97)	284 (23.4)	0.33 (2.28)	274 (24.5)
Physiotherapist visit	3.55 (5.26)	250 (20.6)	3.53 (4.87)	250 (22.4)
Inpatient night in hospital	1.75 (2.75)	231 (19.0)	1.71 (2.99)	215 (19.3)
Day-case attendance	0.28 (0.92)	266 (21.9)	0.25 (0.84)	245 (21.9)
Hospital outpatient appointment	1.40 (2.02)	263 (21.7)	1.47 (3.19)	247 (22.1)
Emergency department attendance	0.12 (0.75)	253 (20.9)	0.11 (1.09)	244 (21.8)

GP, general practitioner; SD, standard deviation.

**Table V.** Total mean costs of healthcare services and bandage cost based on multiply imputed dataset up to 12-month follow-up.

Cost item	Total mean cost, £ (SD)		Mean difference, compression to standard (95% CI)
	Compression bandage (n = 12,130)	Standard bandage (n = 11,170)	
Doctor appointment at GP practice	34.29 (75.07)	33.09 (73.75)	1.20 (-0.71 to 3.11)
Doctor home visit	7.18 (52.04)	7.16 (41.66)	0.02 (-1.19 to 1.24)
Doctor phone appointment	8.87 (31.56)	8.27 (32.95)	0.60 (-0.23 to 1.43)
Nurse appointment at GP practice	3.91 (11.56)	5.47 (22.37)	-1.56 (-2.01 to -1.11)
Community nurse home visit	12.26 (34.98)	13.94 (41.71)	-1.68 (-2.67 to -0.70)
Occupational therapist visit	13.42 (48.47)	16.56 (109.27)	-3.14 (-5.28 to -1.00)
Physiotherapist visit	177.38 (261.03)	176.40 (242.65)	0.99 (-5.50 to 7.47)
Inpatient night in hospital	750.47 (1,170.26)	739.27 (1,248.03)	11.19 (-19.86 to 42.25)
Day case attendance	471.63 (1,527.60)	426.39 (1,407.70)	45.24 (7.42 to 83.06)
Hospital outpatient appointment	206.09 (311.99)	219.26 (453.41)	-13.17 (-23.10 to -3.24)
Emergency department attendance	19.49 (128.53)	18.08 (166.26)	1.41 (-2.38 to 5.22)
Costs associated with bandage*	15.46 (5.50)	5.15 (1.95)	10.30 (10.20 to 10.41)
Bandage itself	7.08 (2.81)	1.61 (0.93)	5.46 (5.41 to 5.52)
Training for compression bandage	2.01 (0.97)	0.04 (0.31)	1.97 (1.95 to 1.99)
Application of bandage	4.47 (1.25)	2.46 (0.60)	2.01 (1.99 to 2.04)
Removal of bandage	1.89 (0.53)	1.04 (0.26)	0.85 (0.84 to 0.86)

* Based on the bandage received by the participant irrespective of which bandage group they were randomized to.

CI, confidence interval; GP, general practitioner; SD, standard deviation.

### Bandage costs

The mean costs associated with bandages were £15.46 in the compression bandage group and £5.15 in the standard bandage group. These costs incorporate crossovers (e.g. where a participant randomized to receive a compression bandage actually received a standard bandage); costs for a standard bandage were applied. A breakdown of the bandage cost components can be seen in Table V.

### Health-related quality of life

Baseline utility levels were very similar across groups, with mean baseline scores of 0.514 (SD 0.215) and 0.511 (SD 0.220) for the compression and standard bandage groups, respectively. At 12 months, mean utilities were 0.705 (SD 0.263) for the compression bandage group and 0.709 (SD 0.255) for the standard bandage groups; hence, utilities remained similar. There was no statistically significant difference in QALYs over the 12 months for all available cases; a mean difference (for compression minus standard) of -0.0027 (95% CI -0.0137 to 0.0082) when controlling for baseline utility, and 0.0003 (95% CI -0.0112 to 0.0118) when controlling for all covariates.

### Base case analysis

Participants in the compression bandage group incurred additional costs; a total mean cost of £1,720 (95% CI £1,678 to £1,763) was incurred per participant in the compression bandage group versus £1,669 (95% CI £1,626 to £1,712) for the standard bandage group, based on the multiply imputed dataset (Table VI). In terms of the EQ-5D-5L findings, QALYs were found to be marginally lower in the compression bandage group (0.602 mean QALYs (95% CI 0.599 to 0.606)) compared to participants in the standard bandage group (0.607 mean QALYs (95% CI 0.603 to 0.610)) (Table VI). The incremental analysis found a mean incremental cost of £52.68 (95% CI £50.56 to £54.80) and a mean reduction of -0.0050 (95% CI -0.0051 to -0.0049) QALYs, based on a bivariate model that used seemingly unrelated regression and adjusting for covariates. The incremental net monetary benefit was -£152.01 (95% CI -£155.30 to -£148.73) at the £20,000 per QALY threshold, and -£201.68 (95% CI -£205.85 to -£197.51) at the £30,000 per QALY threshold, thereby indicating that compression bandages do not represent a cost-effective option when compared to standard bandages.

**Table VI.** Mean costs and quality-adjusted life years by group (multiply imputed dataset).

Item	Mean cost (£); SE (95% CI)	Mean QALYs; SE (95% CI)
Compression bandage	1,720.47; 21.62 (1,678.09 to 1,762.85)	0.602; 0.002 (0.599 to 0.606)
Standard bandage	1,669.06; 21.84 (1,626.26 to 1,711.87)	0.607; 0.002 (0.603 to 0.610)
Difference (compression vs standard)*	51.41; 30.77 (-8.90 to 111.71)	-0.004; 0.003 (-0.009 to 0.001)

* Adjusted for all covariates (baseline utility, sex, age, baseline Oxford Knee Score, and study site).

CI, confidence interval; QALYs, quality-adjusted life years; SE, standard error.

**Fig. 2:**
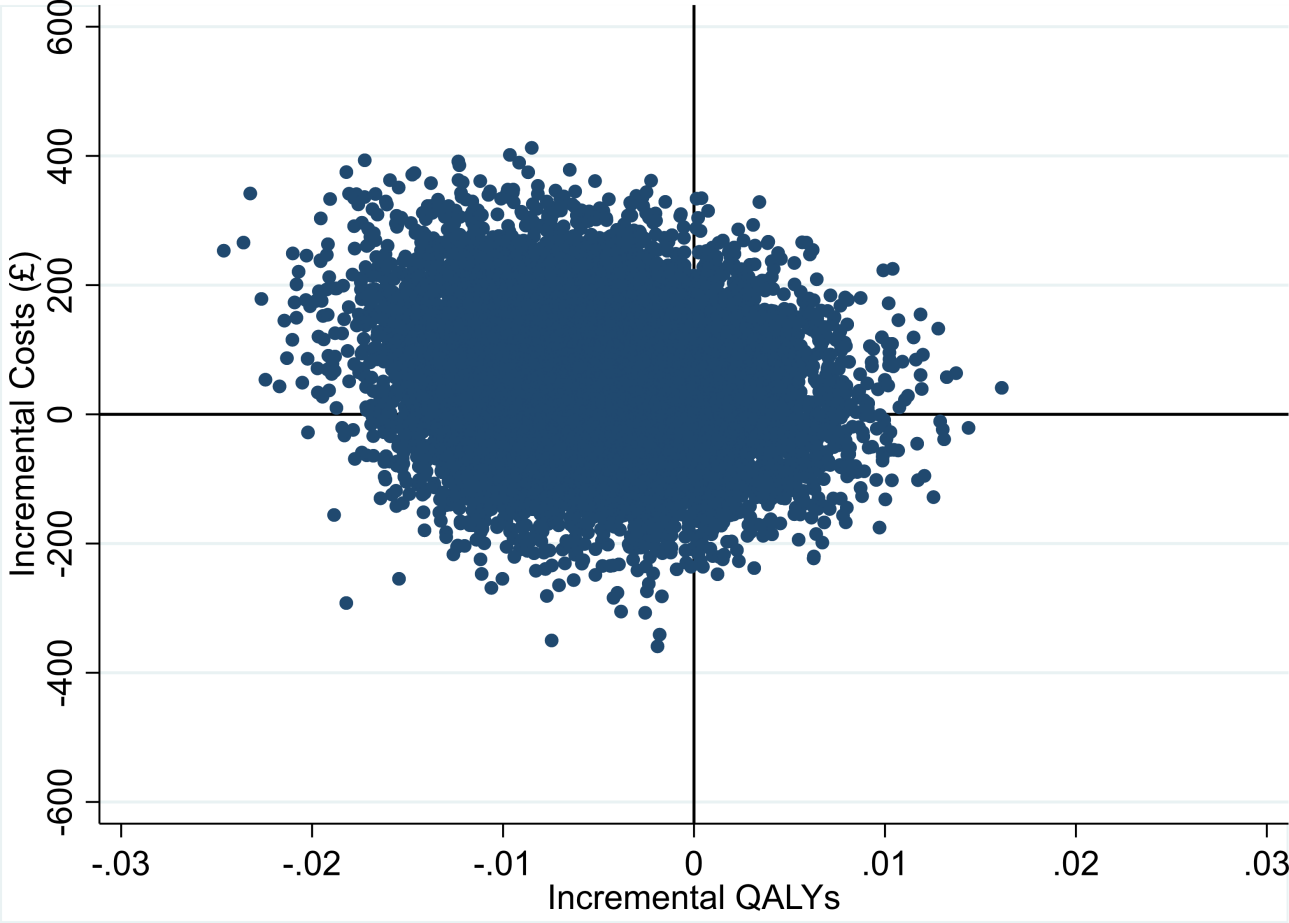
Scatter plot on the cost-effectiveness plane: incremental costs and incremental quality-adjusted life years (QALYs).

**Fig. 3:**
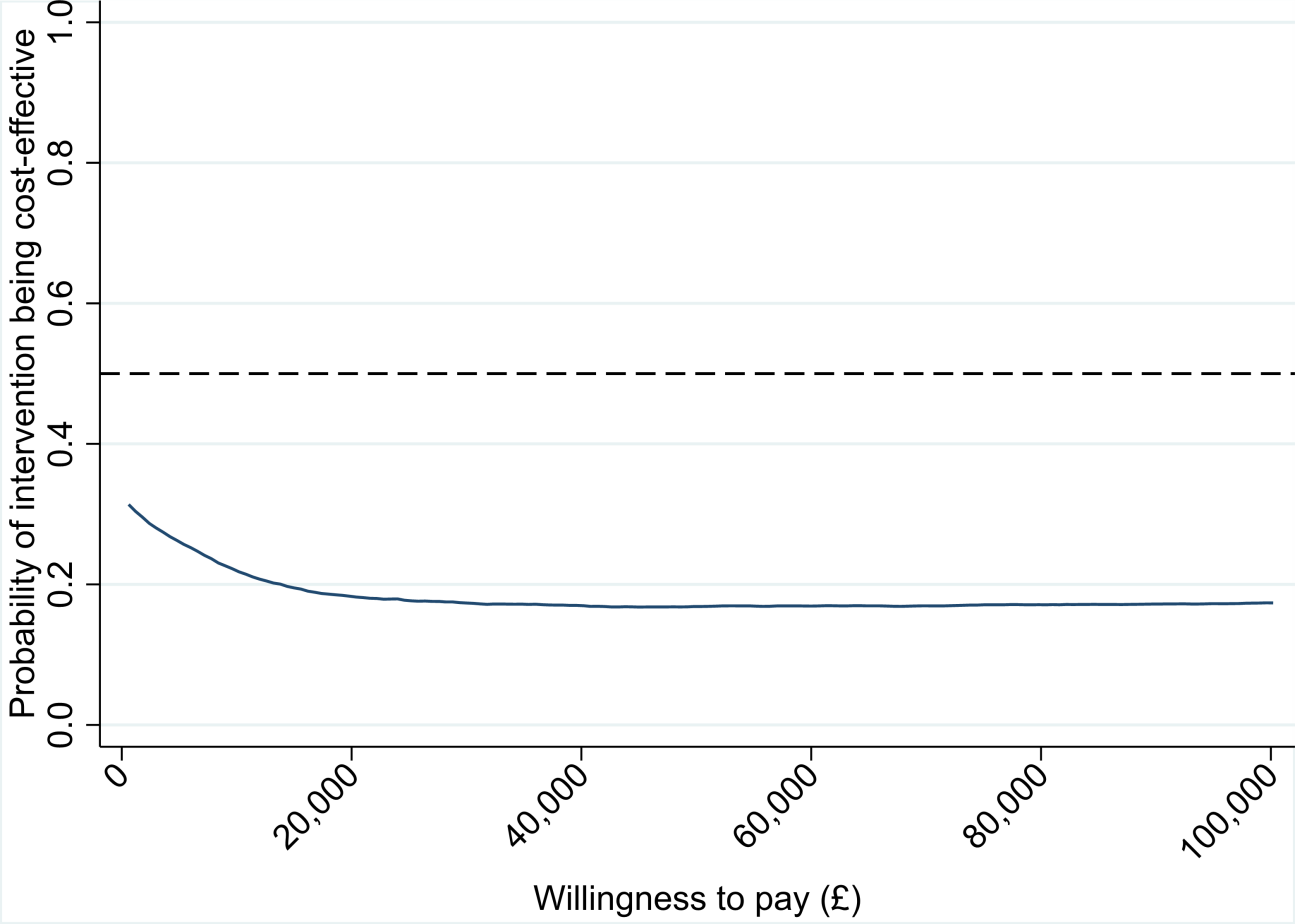
Cost-effectiveness acceptability curve.

Although the cost-effectiveness point estimate indicates that compression bandages are dominated by standard bandages (i.e. the compression bandage was found to be more expensive and less beneficial in terms of QALYs gained), there was considerable uncertainty around this finding, as illustrated by the point estimates of the 10,000 bootstrap sample estimates in Figure 2. Estimates appear in all four quadrants of the cost-effectiveness plane, although more heavily populated in the north-west and south-west quadrants. Figure 3 illustrates the probability of compression bandages being cost-effective for different willingness-to-pay thresholds. The probability of compression bandages being cost-effective for a threshold of £20,000 per QALY is 18.3%, and 17.4% at the £30,000 per QALY threshold.

### Secondary analysis

The secondary analysis (i.e. inclusion of productivity losses) found that the compression bandage group was associated with an additional mean cost of £416 (95% CI 411 to 422) compared with the standard bandage group. This was driven by participants in the compression bandage group encountering more sick leave days than the standard bandage group (23 days (SD 51) versus 18 days (SD 40), based on all available cases; n = 585 in the compression bandage group and n = 507 in the standard bandage group) over 12 months.

### Sensitivity analysis

Table VII summarizes the incremental mean QALYs, incremental mean costs, cost-effectiveness finding (i.e. ICER, or dominated), and probability of compression bandages being cost-effective for the four sensitivity analyses. The cost-effectiveness findings remained robust to the sensitivity analyses undertaken, with the compression bandage dominated by the standard bandage for all four sensitivity analyses. The complete case analysis undertaken for SA1 was the only sensitivity analysis to find that the incremental costs and QALYs were not statistically significant, and the probability of being cost-effective at the £20,000 threshold increased to 29%.

**Table VII.** Summary results for incremental analysis, cost-effectiveness and uncertainty for the base case analysis, secondary analysis, and sensitivity analyses.

Sensitivity analysis	Incremental mean cost, £ (95% CI)*	Incremental mean QALYs (95% CI)*	ICER (£), cost per QALY	Probability cost-effective, £20,000/QALY, %
Base case (MI), NHS perspective	52.68 (50.56 to 54.80)	-0.0050 (-0.0051 to -0.0049)	Dominated†	18
Secondary analysis: societal perspective	416.08 (410.63 to 421.53)	-0.0038 (-0.0039 to -0.0037)	Dominated†	6
SA1: complete case analysis	57.69 (-173.30 to 288.67)	-0.0025 (-0.0154 to 0.0104)	Dominated†	29
SA2: complications costed using PAS data and AE forms	60.86 (58.65 to 63.06)	-0.0036 (-0.0037 to -0.0035)	Dominated†	21
SA3: alternative cost of IP, OP, day case	47.14 (45.45 to 48.53)	-0.0050 (-0.0051 to --0.0049)	Dominated†	13
SA4: initial inpatient stay included (PAS data)	27.34 (24.91 to 29.77)	-0.0044 (-0.0045 to -0.0043)	Dominated†	25

* Difference between groups (compression bandage vs standard bandage), with a bivariate model using seemingly unrelated regression used to estimate 95% CIs. All analyses are adjusted for the following covariates: baseline utility, sex, age, baseline Oxford Knee Score, and study site.

† Dominated: compression bandage dominated by standard bandage, i.e. the compression bandage group incurred additional costs and fewer QALYs.

AE, adverse event; CI, confidence interval; ICER, incremental cost-effectiveness ratio; IP, inpatient; MI, multiple imputation; OP, outpatient; PAS, patient administration system; QALY, quality-adjusted life year; SA, sensitivity analysis.

## Discussion

The base case analysis found compression bandages to be associated with an additional cost of £53 and 0.005 fewer QALYs in comparison to standard bandages. Compression bandaging was therefore dominated, with a 18% probability of the compression bandage being cost-effective at the £20,000 per QALY willingness-to-pay threshold. When productivity losses were incorporated, the dominance finding remained, with a larger cost difference between the groups, and the likelihood of cost-effectiveness lowered. Key cost drivers were participants’ use of hospital services, specifically inpatient stays, and outpatient and day-case attendances. Results remained robust when sensitivity analyses were undertaken. However, for all analyses, only a slight difference in quality of life was seen, and the spread of estimates on the cost-effectiveness plane illustrates the high level of uncertainty in the overall cost-effectiveness findings.

Completion rates for the economic data were reasonably high for both study groups; at baseline 97% had complete data (comprising EQ-5D-5L data only) and 63% had complete data at 12-month follow-up (comprising EQ-5D-5L and resource use over the past 12 months). Such data on resource use and utilities of individuals undergoing a TKA may be of potential use for future studies. The trial aimed to minimize additional (non-routine) data collection as far as possible in order to keep the respondent burden low, and to investigate the feasibility of obtaining data by taking this pragmatic approach. Consequently, we did not gather information about complications, further to the details collected on the adverse event forms and information from PAS data, and the secondary analysis undertaken from the societal perspective only included the impact on lost productivity. This was considered a key cost to capture, while being mindful that the trial aimed to minimize data collection from participants. It is acknowledged, however, that the societal perspective can take a broader approach than this.

Participants were asked to record healthcare use data over the past 12 months; use of such a recall period should be considered when interpreting the results, as it may have had implications for the accuracy of the responses reported by participants. Unsurprisingly, assumptions were necessary during the analysis. For instance, rather than collecting detailed micro-costing information regarding the standard wool and crepe bandage received, all participants in the standard care group were assumed to incur the cost of a Velband bandage (BSN Medical, Germany) plus Hosicrepe bandage (Paul Hartmann, Germany), this being considered representative of the type of standard bandages received by participants, as advised by the clinical study team. Similarly, assumptions were also made for the cost of healthcare professionals involved in applying and removing bandages, and receiving training, based on information provided by those involved in the study.

It is acknowledged that for the sensitivity analysis exploring the inclusion of complication costs using data obtained from PAS and adverse event forms (SA2), there is the possibility of double counting the costs of complications. For example, where a cost was attached for a pulmonary embolism based on PAS data, it may also have been reported in the participant’s questionnaire. We also recognize that the list of complications that data were collected for is not considered exhaustive, but aimed to include the most relevant complications, following clinical advice. Initial inpatient stay for knee arthroplasty obtained from PAS was considered for inclusion in the base case analysis, with the health economic analysis plan specifying that relevant data obtained via PAS or PROMs would feed into the analysis where data allow. However, inclusion of initial inpatient stay data considerably reduced the data available for use in the analysis; 675 participants who had complete baseline and 12-month questionnaire responses but a missing initial inpatient stay response would have been lost from the analysis. The inclusion of initial inpatient stay was therefore explored via sensitivity analysis (SA4), yielding similar findings to the base case.

The results from this analysis add to the evidence base around the cost-effectiveness of bandages for TKA, which to the authors’ knowledge has not been reported previously for compression bandages. In conclusion, there was no evidence of compression bandages being a cost-effective option when compared with standard bandages applied postoperatively following knee arthroplasty surgery. However, the reduction in QALYs was very small, with uncertainty surrounding the overall cost-effectiveness findings.

**Take home message**

- Use of a two-layer compression bandage is marginally less effective in terms of health-related quality of life.

- It is more expensive when compared with a standard bandage following total knee arthroplasty.

- Therefore, it is unlikely to provide a cost-effective option.

## Data Availability

The data that support the findings for this study are available to other researchers from the corresponding author upon reasonable request.
